# CREB3L1 Modulates Extracellular Matrix Gene Expression and Proliferation in Glaucomatous Lamina Cribrosa Cells

**DOI:** 10.3390/biomedicines14030633

**Published:** 2026-03-11

**Authors:** Mustapha Irnaten, Ellen Gaynor, Liam Bourke, Colm O’Brien

**Affiliations:** 1 Clinical Research Centre, School of Medicine, University College Dublin, D04 V1W8 Dublin, Ireland; ellegaynor96@gmail.com (E.G.); cobrien@mater.ie (C.O.); 2 Department of Ophthalmology, Mater University Hospital, D07 R2WY Dublin, Ireland; liambourke1@gmail.com

**Keywords:** glaucoma, fibrosis, lamina cribrosa cells, CREB3L1

## Abstract

**Background:** Fibrotic remodelling of the lamina cribrosa (LC) is a defining pathological feature of glaucomatous optic neuropathy and contributes to progressive optic nerve head deformation and axonal vulnerability. LC cells from glaucomatous donors exhibit a myofibroblast-like phenotype characterised by excessive extracellular matrix (ECM) production, a process associated with chronic cellular stress. cAMP responsive element-binding protein 3-like 1 (CREB3L1) is an endoplasmic reticulum–resident transcription factor implicated in stress-responsive regulation of collagen synthesis and matrix homeostasis. The role of CREB3L1 in glaucomatous LC cells, however, remains poorly defined. **Methods:** Primary human LC cells derived from donors with confirmed glaucoma (GLC; n = 3) and age-matched non-glaucomatous controls (NLC; n = 3) were examined. CREB3L1 expression was assessed at the mRNA and protein levels using quantitative RT-PCR and Western immunoblotting. The functional effects of CREB3L1 suppression were evaluated using siRNA-mediated knockdown in GLC cells, followed by analysis of ECM gene transcription (α-smooth muscle actin, collagen type I alpha 1, fibronectin) and cellular metabolic activity using an MTS assay. **Results:** CREB3L1 mRNA and protein expression were significantly elevated in GLC cells compared with NLC cells. siRNA-mediated knockdown of CREB3L1 effectively reduced its expression in GLC cells and was associated with significant suppression of profibrotic ECM gene transcription. In addition, CREB3L1 knockdown resulted in a marked reduction in cellular metabolic activity in glaucomatous LC cells. **Conclusions:** These findings identify CREB3L1 as a regulator of ECM-associated gene expression and cellular behaviour in glaucomatous lamina cribrosa cells. While preliminary, the data suggest that CREB3L1 may contribute to pathological fibrotic remodelling at the optic nerve head. Further mechanistic and in vivo studies will be required to determine whether modulation of CREB3L1-mediated pathways represents a viable therapeutic strategy in glaucoma.

## 1. Introduction

Glaucoma is the leading cause of irreversible yet potentially preventable blindness worldwide, affecting more than 75 million individuals and projected to increase substantially over the coming decades [1]. It is a chronic, progressive optic neuropathy characterised by excavation and pallor of the optic nerve head (ONH), accompanied by the gradual loss of retinal ganglion cells (RGCs) and corresponding visual field defects [2]. Although elevated intraocular pressure (IOP) remains the principal modifiable risk factor, increasing evidence suggests that structural and molecular events occurring at the ONH are central determinants of disease susceptibility and progression [3,4].

A defining pathological feature of glaucomatous optic neuropathy is excessive remodelling of the extracellular matrix (ECM) within the lamina cribrosa (LC), the principal load-bearing structure of the ONH [3,5]. Histological and ultrastructural studies have demonstrated marked fibrosis of the LC connective tissue in glaucoma, characterised by abnormal collagen deposition, elastin remodelling, and increased tissue stiffness [5,6]. These changes are driven, at least in part, by profibrotic signalling pathways including transforming growth factor beta (TGFβ) [7], and are exacerbated by chronic mechanical strain imposed by elevated IOP [4]. Progressive ECM accumulation and altered biomechanical properties of the LC compromise its ability to support traversing RGC axons, rendering them vulnerable to deformation, compression, and impaired axoplasmic transport [2,8]. Despite these observations, the molecular mechanisms linking cellular stress responses to fibrotic remodelling at the ONH remain incompletely defined.

Fibrosis is broadly defined as the pathological overproduction and accumulation of ECM components, leading to disruption of normal tissue architecture and function [9]. In glaucoma, fibrotic remodelling is not confined to the trabecular meshwork but is also prominent within the LC, where resident fibroblast-like cells undergo phenotypic transformation toward a myofibroblast-like state [2,5]. This transition is characterised by increased expression of contractile proteins such as α-smooth muscle actin (αSMA), along with heightened synthesis of structural ECM proteins including collagen type I and fibronectin [5,10]. These biosynthetic demands place a substantial burden on the endoplasmic reticulum (ER), implicating ER stress and unfolded protein response (UPR) signalling as potential contributors to glaucomatous pathology [11,12,13,14,15].

Emerging evidence supports a role for oxidative stress and ER stress across multiple ocular tissues implicated in glaucoma, including the trabecular meshwork, retinal ganglion cells, and the ONH [11,12,13,14,15,16,17,18,19,20,21,22,23]. Previous work from our group has demonstrated mitochondrial dysfunction, calcium dysregulation, and oxidative stress in human glaucomatous LC cells, collectively suggesting chronic perturbation of intracellular homeostasis [24,25,26]. However, the transcriptional regulators that couple ER stress signalling to pathological ECM production in LC cells remain poorly characterised.

cAMP responsive element-binding protein 3-like 1 (CREB3L1), is a member of the CREB3 subfamily of basic leucine zipper (bZIP) transcription factors that function as ER-resident stress sensors [27,28]. Structurally and mechanistically related to activating transcription factor 6 (ATF6), CREB3L1 is synthesised as an inactive transmembrane protein anchored in the ER [28]. Under conditions of ER stress, CREB3L1 undergoes regulated intramembrane proteolysis, releasing its N-terminal transcriptionally active fragment, which translocates to the nucleus and drives expression of genes involved in proteostasis, secretion, and ECM production [29,30].

Beyond its canonical role in ER stress responses, CREB3L1 has been implicated in connective tissue biology, chondrogenesis, and fibrosis [31,32]. Notably, collagen type I alpha 1 (Col1A1) is a well-established downstream transcriptional target of CREB3L1, linking ER stress signalling directly to ECM synthesis [31]. In glaucomatous LC cells, where elevated intracellular calcium and chronic stress signalling coexist with excessive ECM gene expression [2,5,26], CREB3L1 represents a compelling candidate regulator at the intersection of stress adaptation and fibrotic remodelling.

Recent work has further highlighted the relevance of collagen dysregulation to glaucoma through insights from osteogenesis imperfecta, a heritable collagen disorder predominantly caused by mutations in Col1A1 [33]. Individuals with osteogenesis imperfecta exhibit altered ocular biomechanics, including changes in trabecular meshwork resistance, corneal properties, and lamina cribrosa compliance, which may confer increased vulnerability to glaucomatous damage independent of IOP [33,34,35]. Given that CREB3L1 directly regulates Col1A1 transcription, these converging lines of evidence suggest that CREB3L1-mediated pathways may play a broader role in shaping the biomechanical and fibrotic landscape of the glaucomatous ONH [31,36,37,38].

In this study, we investigated the expression and functional role of CREB3L1 in human lamina cribrosa cells derived from glaucomatous and non-glaucomatous donors. Using siRNA-mediated knockdown, we examined the impact of CREB3L1 suppression on ECM gene transcription and cellular metabolic activity, aiming to clarify whether CREB3L1 contributes to the profibrotic phenotype characteristic of glaucomatous LC cells.

## 2. Materials and Methods

### 2.1. Cell Culture and Characterisation

Primary cultures of human lamina cribrosa (LC) cells were established from age-matched donor eyes without glaucoma (NLC, n = 3; mean age 77.8 ± 6.38 years) and from donor eyes with clinically confirmed glaucoma (GLC, n = 3; mean age 81.0 ± 10.23 years). All LC cells used in this study were obtained from the Central Florida (Tampa) Eye Bank for Transplant and Research within 24 h post-mortem (Table 1). Glaucoma diagnosis was confirmed based on available medical records and/or documentation of a prior glaucoma diagnosis provided by family members. All human tissue was obtained and managed in accordance with the Declaration of Helsinki for research involving human tissue.

The methods used to isolate LC cells from human optic nerve head tissue were originally described by Lambert [39] and subsequently refined by Lopez et al. [40]. Primary LC cultures were characterised following expansion by positive immunostaining for α-smooth muscle actin (α-SMA) and negative staining for glial fibrillary acidic protein (GFAP) and ionised calcium-binding adaptor molecule 1 (Iba1), confirming a fibroblast-like phenotype and excluding astrocytic and microglial contamination [10].

LC cells were cultured in Dulbecco’s Modified Eagle Medium (DMEM; Sigma, Dublin, Ireland) supplemented with 10% (*v*/*v*) heat-inactivated foetal bovine serum (FBS), 1% L-glutamine, and 1% penicillin–streptomycin. Cells were maintained at 37 °C in a humidified atmosphere containing 5% CO_2_. Cells were cryopreserved in liquid nitrogen and thawed as required. Following thawing, cells were seeded at high density and expanded to 80–90% confluence prior to passaging using trypsin/EDTA (2–5 min at 37 °C). All experiments were performed using cells between passages 3 and 8. Prior to siRNA transfection, LC cells were serum-deprived for 24 h.

### 2.2. LC Cell Transfection with Anti-CREB3L1 siRNA

CREB3L1-specific small interfering RNAs (siRNAs) were obtained as a pooled SMARTpool consisting of four distinct siRNA sequences, along with a non-targeting negative control siRNA (siCONTROL), purchased from Dharmacon (Research Inc., Dublin, Ireland). LC cells were seeded in six-well plates at a density of 1 × 10^6^ cells/mL (three wells per condition) and transfected at 40–50% confluence using a lipid-based transfection reagent (Dharmacon), according to the manufacturer’s instructions.

The four CREB3L1 siRNA sequences used were as follows: GAAAGUCCAUCCUGUAUGU; GAAAAUCCGUUCUAAAGUA; UUACUGCCGUGACGAGAUU; and CGUCUACCUUCACCAAUAU. Prior to transfection, cells were cultured for 48 h, followed by serum and antibiotic deprivation in Opti-MEM^®^ medium for 24 h. Transfection was performed using 50 nM CREB3L1 siRNA or 50 nM non-targeting control siRNA in Opti-MEM^®^ medium without antibiotics. After 6 h, the transfection, medium was replaced with complete growth medium containing FBS and antibiotics, and cells were incubated for a further 68 h.

All siRNA sequences were validated in silico to minimise off-target effects. Transfection efficiency was assessed using Spotiflow “SliGlow” fluorescent labelling, yielding an efficiency of approximately 80–91%. To elucidate the transfection related cytotoxicity, we transfected LC cells with varying concentrations of CREB3L1 siRNA (untreated, 1 nM, 10 nM, 50 nM, 100 nM. CREB3L1 and ECM gene expression levels were subsequently quantified by quantitative RT-PCR using the ΔΔCt method, normalised to the housekeeping gene 18S rRNA. All transfection experiments were performed with three technical replicates per donor and repeated across three independent donors per group (n = 3 NLC and n = 3 GLC).

### 2.3. RNA Extraction, cDNA Synthesis & Quantitative Real-Time RT-PCR

Total cellular mRNA was isolated from confluent T75 flasks by addition of Tri-Reagent solution which acts to perforate the cells. The resulting solution was transferred to a 1.5 mL Eppendorf tube and 100 μL chloroform (per 1 mL Tri-Reagent) was added. The tube was subsequently mixed well and placed on ice for ~5 min. Following incubation on ice, the sample was centrifuged at 12,000 *g* at 4 °C for 12 min to enable phase separation. With great caution, the uppermost, aqueous phase was transferred to a new Eppendorf tube. This aqueous layer contains the mRNA, and 500 μL isopropanol was then added and the mixture left to incubate on ice for 5 min, before it was spun in a centrifuge at 12,000 *g* for 8 min at 4 °C. Subsequently, the supernatant was aspirated and discarded, while the pellet was spun in the centrifuge at 7500 *g* for 5 min at 4 °C with 1 mL 70% ethanol to purify the pellet. The supernatant was removed and discarded, while the RNA pellet was left to air dry before it was resuspended in 12–15 μL of diethylpyrocarbonate-treated (DEPC) water and stored at −80 °C.

The isolated mRNA was subsequently reverse-transcribed into complimentary DNA (cDNA), its essential form for RT-PCR. This procedure involved the mixture of 2 μL corresponding mRNA with 13.5 μL DEPC water, 1 μL deoxynucleotides (dNTPs), 1 μL oligo dT and 2 μL 10x AMV reverse transcriptase buffer. When all components were combined in a 0.2 mL PCR Eppendorf tube, the mixture was heated at 70 °C for 2 min and then put on ice for 1–2 min before 0.5 μL AMV reverse transcriptase was added. A thermocycler was used to conduct the reverse transcription reaction. The programme selected involved a 90 min cycle at 45 °C, two minutes at 90 °C, followed by a cooling stage down to 4 °C. The resulting cDNA was stored at −80 °C. The concentration and the purity of the RNA were determined using a NanoDrop™ 1000 Spectrophotometer (Thermo Scientific, Dublin, Ireland).

Quantitative RT-PCR was carried out on a LightCycler^®^ 480 System (ROCHE Diagnostics, Dublin, Ireland), using Platinum^®^ SYBR^®^ Green qPCR Super-Mix (ROCHE, Dublin, Ireland) along with human gene-specific forward and reverse primers (Table 1). The mixture in each well, of the 96-well plates used, comprised 4.25 μL DEPC water, 6.25 μL SYBR Green PCR Master Mix, 1 μL of cDNA and 0.5 μL of each corresponding primer (forward and reverse). To normalise CT values, the ribosomal RNA 18S was utilised as an internal housekeeping control gene. The PCR primer sequences are displayed in Table 2. The PCR amplification cycle selected firstly denatured samples at 95 °C for 10 min, followed by 45 cycles of another denaturation at 95 °C for 10 s, primer annealing at 55 °C for 30 s, elongation at 72 °C for 30 s and final elongation at 72 °C for 5 min. The relative fold change in gene expression for each PCR product was evaluated using the 2ΔΔCt equation [41].

### 2.4. Protein Extraction and Western Blot Analysis

Once treated, cells were washed twice with ice-cold phosphate-buffered saline (PBS) (Sigma, Ireland) solution, collected in PBS, and then centrifuged at 1200 *g* for 5 min at 4 °C. Note that the PBS being used for washing the cells was iso-osmotically adjusted (Dulbecco’s Modified PBS, ~300 mOsm) to prevent the cells from lysis. The resulting supernatant was discarded, while the pellet was treated with radioimmunoprecipitation assay (RIPA) buffer containing protease/phosphatase inhibitor cocktail (Sigma, Dublin, Ireland). This treatment resulted in the collection of crude cell lysate that was then incubated on ice for 10 min before the cells were centrifuged at 15,000 *g* for 10 min at 4 °C. The clear supernatant was collected, and protein concentrations calculated through use of the Bradford assay.

Electrophoresis of the protein extracts (20 μg/lane) was performed on 10% polyacrylamide-SDS gels and resulting proteins were transferred to a nitrocellulose membrane. Membranes were blocked for 1 h at room temperature with 3% fat-free milk in Tris-buffered saline with 0.1% Tween-20 (TBST), followed by overnight incubation at 4 °C with CREB3L1 primary antibody (Table 3). Membranes were then washed with TBST and incubated for one hour at room temperature with the secondary antibody conjugated to horseradish peroxidase (Table 3). The membranes were re-probed with anti-*β*-actin antibody (Table 3), as a control to ensure equal protein loading. The conditions of incubations of *β*-actin are similar to CREB3L1 primary antibody. Standard protocols were employed to process the protein blots using enhanced chemiluminescence detection reagents (Fisher Scientific, Dublin, Ireland) and for their ultimate analysis with Image J software 1.54 (NIH, LOCI, University of Wisconsin, USA) with data normalised to *β*-actin.

### 2.5. Cell Proliferation: MTS Assay

The proliferation of human primary LC cell cultures was evaluated using the 3-(4,5-dimethylthiazol-2-yl)-5-(3-carboxy-methoxyphenyl)-2-(4-sulfophenyl)-2H-tetrazolium (MTS) assay according to the manufacturer’s protocol. Cells (10^4^ cells/well) (n = 3 donors per group) were plated in triplicate into 96-well plates in full growth medium (DMEM-F12) for 48 h. The medium was then removed, and cells were incubated overnight in serum-free medium prior to assignment into untreated (control) or treated wells.

Cells were treated with anti-CREB3L1 siRNA (50 nM) for 6 h in antibiotic- and serum-free medium, after which cells were cultured for an additional 48 h in full growth medium. CellTiter^®^ 96 AQueous One Solution Reagent (20 μL) (Promega, Dublin, Ireland) was added to each well, followed by incubation for a further 3 h. Absorbance was measured at 490 nm using a SpectraMax spectrophotometer (Molecular Devices Corp., Wokingham, UK) and analysed using SoftMax Pro 7.1.1 software (Molecular Devices Corp., Wokingham, UK).

Mean values and standard deviation (SD) were calculated for each condition. Proliferation was expressed relative to untreated controls using the formula:

Proliferation = (*OD*_siRNA_ − *OD*_*Blank*_)/(*OD*_*Blank*_ − *OD*_*Blank*_).

### 2.6. Statistical Analysis

Three normal eye donors (controls; NLC, n = 3) and three glaucoma eye donors (GLC, n = 3) were used in this study. Data analysis was performed using Origin version 7.0 software (OriginLab, Bucks, UK) and results are presented as mean ± standard deviation. Statistical analyses were performed at the biological replicate (donor) level using donor-averaged values. Comparisons between two groups were performed using a two-tailed unpaired Student’s t-test, where appropriate. For comparisons involving more than two groups, one-way analysis of variance (ANOVA) followed by Tukey–Kramer post hoc testing was applied. Statistical significance was set at *p* < 0.05 and is denoted by * *p* < 0.05. In all analyses, “n” indicates the number of independent eye donors.

## 3. Results

### 3.1. CREB3L1 Gene Transcription Is Elevated in LC Cells from Glaucomatous Donors

**Figure 1:**
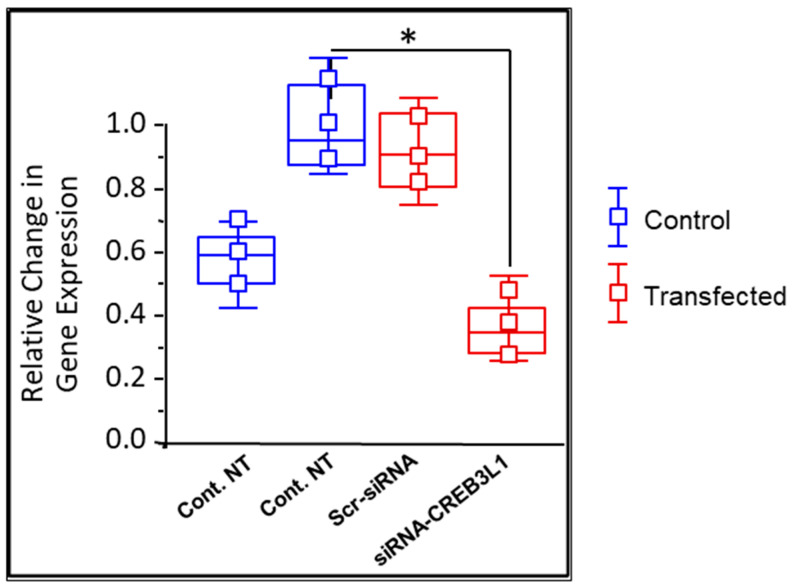
Comparison of LC cells from normal and glaucomatous age-matched donors, under varying treatment conditions, with respect to CREB3L1 gene expression levels. Quantitative Real-Time PCR shows gene expression levels of CREB3L1 in NLC cells versus GLC cells, normalised to the housekeeping gene 18s. Note that values that demonstrate a significant difference to the control are denoted by asterisks (* *p* < 0.05 vs. Ctrl). There is a significant elevation in CREB3L1 mRNA in GLC cells (0.99 ± 0.062) compared to NLC cells (0.636 ± 0.05) (n = 3; *p* < 0.05). No significant change in gene expression is shown at GLC basal conditions (Ctrl) versus GLC cells treated with “scramble” siRNA (scr-siRNA), which works to ensure effective sequence-specific silencing with siRNA-CREB3L1. Moreover, this quantitative real-time PCR demonstrates a significant reduction in CREB3L1 mRNA in GLC cells treated with siRNA-CREB3L1 (0.372 ± 0.068) versus CREB3L1 mRNA levels in untreated GLC cells (0.99 ± 0.062).

**Figure 2:**
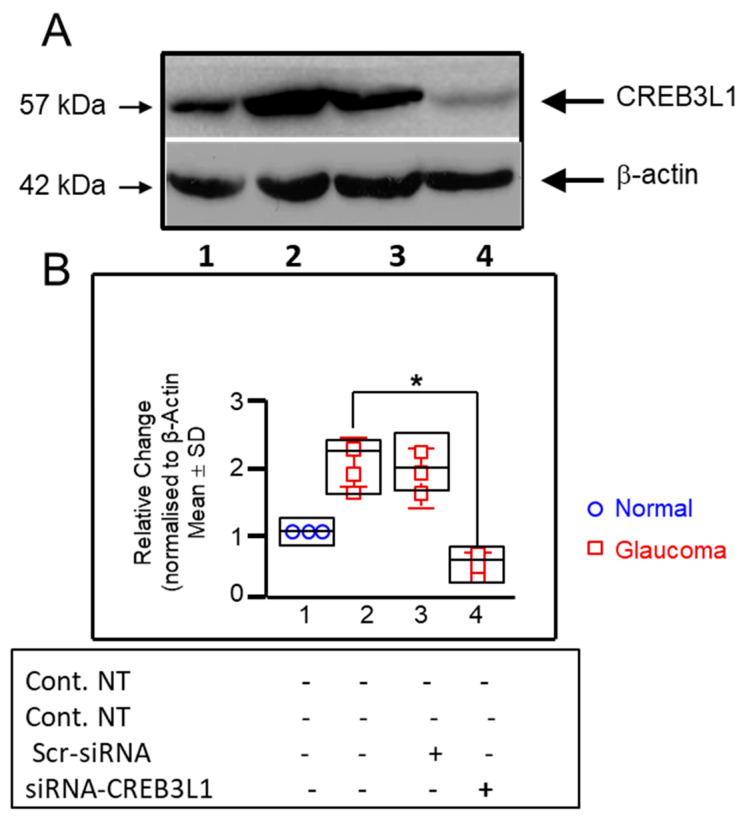
Western blot demonstrating differential total-CREB3L1 protein levels between LC cells from normal (lane 1) versus glaucomatous (lanes 2–4) donors, in the presence (+) and absence (-) of “scramble” Scr-siRNA (negative control) and CREB3L1-specific siRNA. (**A**) Western immunoblotting analysis of CREB3L1 in LC cell lysates in normal and glaucoma LC cells, subject to varying treatments. To confirm equal concentrations of proteins loaded into each lane (20 μg), an additional step of washing and reblotting of membranes with β-actin antibody was performed in the experiment (Ctrl). (**B**) Results are presented as mean ± SD from three normal and three glaucoma LC cell donors. Total CREB3L1 protein expression was significantly increased in basal GLC cells compared to NLC cells (2.062 ± 0.312-fold vs. 1.0; n = 3; *p* < 0.05). No significant difference was observed between untreated GLC cells and GLC cells treated with scramble siRNA. CREB3L1-specific siRNA treatment significantly reduced total CREB3L1 protein levels compared to untreated GLC cells (0.272 ± 0.093-fold vs. 2.062 ± 0.312-fold; n = 3; * *p* < 0.05).

Quantitative RT-PCR allowed for the comparison of CREB3L1 mRNA levels in GLC cells and age-matched NLC cells. The difference in levels was expressed as a fold change relative to normalised ribosomal RNA 18S. CREB3L1 gene transcription was significantly elevated, in GLC cells versus NLC cells, 0.99 ± 0.062 versus 0.636 ± 0.05 (n = 3; *p* < 0.05; Figure 1). This was further reinforced through Western blotting for CREB3L1 protein. In this experiment, GLC cells were shown to have higher levels of CREB3L1 protein than age-matched NLC control cells (0.542 ± 0.069 vs. 0.334 ± 0.047 × 10^3^ a.u., n = 3; *p* < 0.05; Figure 2B).

### 3.2. CREB3L1 Expression Is Inhibited by Anti-CREB3L1 siRNA in GLC Cells

We next examined the effect of CREB3L1 siRNA knock-down on CREB3L1 gene expression in GLC cells. We found a significant gene reduction in CREB3L1 in siRNA-CREB3L1-treated GLC cells compared to untreated GLC cells, 0.372 ± 0.068 versus 0.99 ± 0.062 (n = 3; *p* < 0.05; Figure 1). At the protein expression levels, Western blotting showed a significant drop in CREB3L1 levels in treated GLC cells versus untreated GLC cells (0.272 ± 0.093 vs. 2.062 ± 0.312, n = 3; *p* < 0.05; Figure 2B).

### 3.3. ECM Genes Transcription Is Elevated in GLC Cells

**Figure 3:**
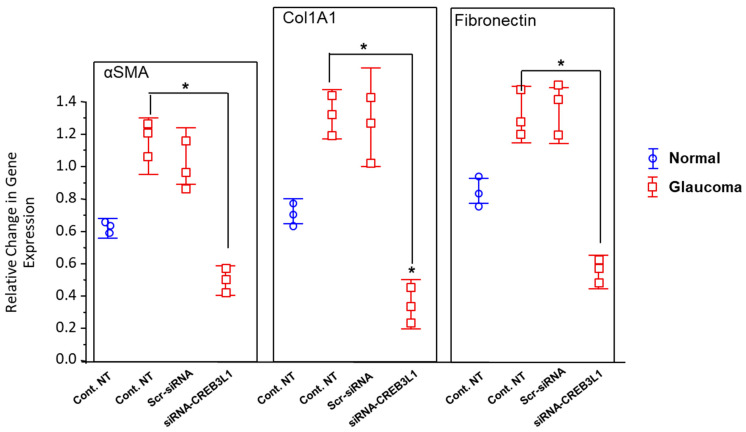
Comparison of the differential transcription of ECM genes, *αSMA*, *Col1A1*, and fibronectin, in NLC cells versus GLC cells versus siRNA-treated GLC cells. Gene expression results of this quantitative RT-PCR are normalised to the housekeeping gene 18s and values that demonstrate a significant difference to the control are denoted by asterisks (* *p* < 0.05 vs. Ctrl). Note that transcription levels of αSMA are all significantly upregulated in GLC cells compared to age-matched NLC cells (n = 3; *p* < 0.05). No significant change in gene expression was demonstrated at GLC basal conditions (Ctrl) versus GLC cells treated with “scramble” siRNA (scr-siRNA), verifying effective sequence-specific silencing with siRNA-CREB3L1. Furthermore, the results of this RT-PCR demonstrate a remarkable suppression of the ECM genes αSMA, Col1A1, and Fibronectin in GLC cells treated with CREB3L1 specific siRNA.

Further rounds of quantitative RT-PCR analysed and compared the differential expression of ECM genes in GLC and age-matched NLC cells, with the difference expressed as a fold change relative to normalised ribosomal RNA 18S. The pro-fibrotic upregulation of well-known ECM genes, including those examined here (α-SMA, Col1A1 and Fibronectin), in GLC cells is a well-known feature of the glaucomatous phenotype. The foremost results of these experiments reinstate this evidence as we have demonstrated that the gene expression of α-SMA, Col1A1 and Fibronectin is significantly elevated in the GLC cells compared to the age-matched NLC cells used in this study (Figure 3).

### 3.4. Anti-CREB3L1 siRNA Inhibited the ECM Gene Expression in GLC Cells

Because of the central role that LC cells play in ECM production in the glaucoma ONH, inherent to the pathophysiology of glaucoma, we studied the effect of CREB3L1 siRNA knockdown on the ECM genes transcription in GLC cells. Across each ECM gene analysed, we found that siRNA-CREB3L1 treatment in GLC cells elicits a remarkably significant transcriptional suppression of these pro-fibrotic genes. *αSMA* transcription was significantly inhibited, in treated GLC versus un-treated GLC cells, 0.507 ± 0.052 versus 1.116 ± 0.193 (n = 3; *p* < 0.05; Figure 3). *Col1A1* transcription was significantly inhibited in treated GLC versus un-treated GLC cells, 0.375 ± 0.066 versus 1.362 ± 0.114 (n = 3; *p* < 0.05; Figure 3). And finally, *fibronectin* transcription was significantly repressed in treated GLC versus un-treated GLC cells, 0.578 ± 0.08 versus 1.364 ± 0.119 (n = 3; *p* < 0.05; Figure 3). These data comprise the average of 3 normal eye donors and 3 glaucoma eye donors. These findings reflect transcriptional changes in ECM-associated genes as assessed by quantitative RT-PCR. Protein-level validation of these targets was not performed in the present study and will be required to determine the extent to which these transcriptional changes translate into functional alterations in extracellular matrix composition.

### 3.5. Anti-CREB3L1 siRNA Inhibited Proliferation in Glaucoma LC Cells

**Figure 4:**
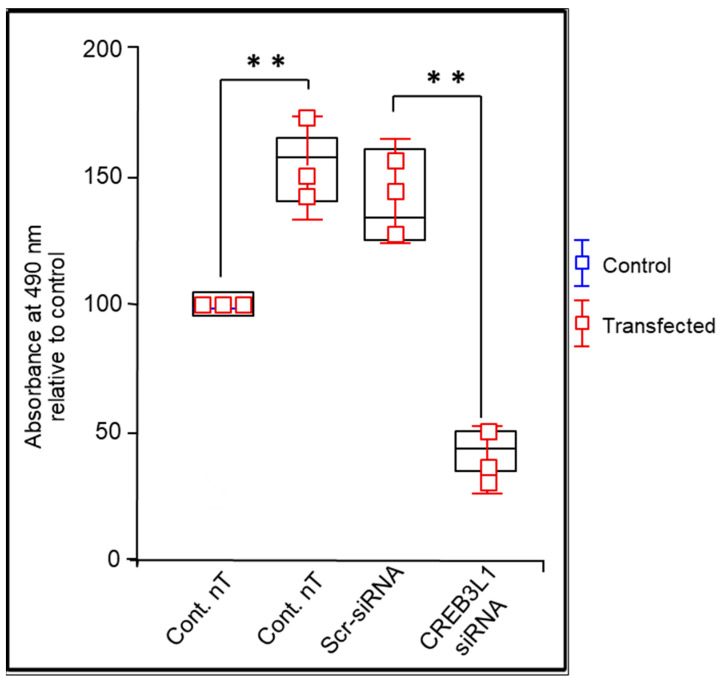
Effect of anti-CREB3L1 siRNA-knockdown on metabolic activity in glaucomatous LC cells. Metabolic activity was assessed by the colorimetric MTS assay. NLC and GLC cells were seeded at equal densities of 1 × 10^6^ cells/mL (3 wells per condition) and transfected at 50–60% confluence with transfection reagent (5 μL). Glaucoma LC cells were cultured for 48 h prior to transfection, then treated with anti-CREB3L1 siRNA for an additional 48 h (see Methods Section 2 for details). Glaucoma LC cells show a significant increase in metabolic activity when compared to normal control LC cells after 96 h of incubation (pre- and post-transfection). MTS assay showed that metabolic activity was significantly increased in GLC cells compared to NLC cells at 96 h (from 98.2 ± 10.1% to 165.6 ± 22.3%, (*p* < 0.01, n = 3), while anti-CREB3L1 siRNA transfection of glaucoma LC cells significantly reduced the metabolic activity (from 165.6 ± 22.3% to 44.4 ± 5.7%), (n = 3; ** *p* < 0.01, 1-way ANOVA with Tukey–Kramer post-test). Values which are significantly different to controls are denoted by asterisks. ** *p* < 0.01 vs. control.

To evaluate metabolic activity, NLC and GLC cells were cultured in standard conditions and either transfected with anti-CREB3L1 siRNA or not (untreated controls). At day four (96 h), the metabolic activity of NLC was 98.2 ± 10.1% and GLC cells was 165.6 ± 22.3%. To examine the effect of anti-CREB3L1 siRNA-knockdown on cell metabolic activity in glaucoma LC cells, we applied 50 nM siRNA to the cells for a total incubation of 48 h. The MTS assay results showed that anti-CREB3L1 siRNA transfection significantly inhibited glaucoma cell metabolic activity after 96 h of total incubation (from 165.6 ± 22.3% to 44.4 ± 5.7%), while scrambled siRNA transfection had no significant effect on GLC metabolic activity (*p* < 0.05, n = 3), (Figure 4).

## 4. Discussion

This study provides evidence that CREB3L1 is upregulated in human lamina cribrosa cells derived from glaucomatous donors and that its suppression attenuates key features of the profibrotic cellular phenotype. Both transcript and protein analyses demonstrated significantly higher basal CREB3L1 expression in glaucomatous LC cells compared with age-matched non-glaucomatous controls, consistent with prior observations implicating chronic ER stress signalling in glaucomatous tissues [12,13,14,15,16,17,18,19,20,21,22,23]. Effective siRNA-mediated knockdown confirms that CREB3L1 is actively expressed and amenable to targeted modulation in this disease-relevant cell population.

Functionally, CREB3L1 silencing resulted in marked suppression of canonical ECM-associated genes, including αSMA, Col1A1, and fibronectin. These molecules are central to the acquisition and maintenance of a myofibroblast-like phenotype and are known contributors to pathological ECM accumulation within the glaucomatous ONH [2,5,10]. The specificity of this effect is supported by the absence of significant transcriptional changes following treatment with scrambled siRNA, indicating that the observed responses are not attributable to non-specific transfection-related stress.

CREB3L1 is activated through regulated intramembrane proteolysis during ER stress, liberating an N-terminal transcription factor that localises to the nucleus and binds conserved response elements within target gene promoters [30,32]. Through this mechanism, CREB3L1 coordinates adaptive responses to increased secretory demand, including upregulation of ECM proteins [31,32]. In the context of glaucomatous LC cells, which exhibit sustained metabolic and biosynthetic stress [11,24,25,26], CREB3L1 activation may represent an adaptive response that becomes maladaptive when chronically engaged, driving excessive matrix production and tissue stiffening [4,5].

The broader biological significance of ER stress and UPR activation has been well documented across diverse pathological contexts, including neurodegeneration, fibrosis, and malignancy [42,43]. The ER serves as a central hub for protein folding, lipid synthesis, calcium storage, and metabolic integration, rendering it highly sensitive to perturbations in cellular homeostasis [43,44]. Disruptions in calcium handling, oxidative balance, or protein load can precipitate ER stress, triggering the UPR to restore equilibrium [45,46,47,48]. However, prolonged or unresolved ER stress can instead promote pathological gene expression programmes, including those governing fibrosis and aberrant cell survival [48,49].

The suppression of ECM gene transcription following CREB3L1 knockdown supports the hypothesis that CREB3L1 acts as a transcriptional driver of fibrotic remodelling in glaucomatous LC cells. Excessive ECM deposition within the LC is known to alter ONH biomechanics, increasing tissue rigidity and impairing axonal support, thereby exacerbating RGC vulnerability [4,19,50]. Modulating transcriptional regulators that lie upstream of matrix production may therefore offer a strategy to mitigate structural damage at the ONH without directly targeting IOP.

In addition to its effects on ECM gene expression, CREB3L1 knockdown significantly reduced metabolic activity as measured by MTS assay. While this assay does not distinguish between reduced proliferation, altered metabolic state, or increased cell death, the findings are consistent with the established role of ER stress signalling in regulating cell cycle progression and survival [15,21,22]. In fibrotic conditions, myofibroblast persistence and expansion contribute to progressive tissue remodelling, and CREB3L1 may participate in sustaining this activated cellular state [31,32].

The link between CREB3L1 and Col1A1 expression also aligns with observations from connective tissue disorders such as osteogenesis imperfecta, in which CREB3L1 dysfunction results in impaired collagen biosynthesis and skeletal fragility [37,38]. These parallels reinforce the concept that CREB3L1 functions as a context-dependent regulator of collagen homeostasis, capable of driving either adaptive or pathological outcomes depending on the duration and intensity of ER stress signalling [31,36].

Several limitations of the present study should be acknowledged. The use of a small number of donor-derived cell lines reflects the inherent challenges of human tissue-based research and underscores the need for cautious interpretation. Furthermore, while our findings establish an association between CREB3L1 expression and fibrotic gene regulation, direct assessment of upstream ER stress markers and CREB3L1 activation dynamics would further strengthen mechanistic insight [12,13,14,15]. Validation at the protein level for downstream ECM targets and in vivo confirmation will be essential to determine the translational relevance of these observations. Moreover, while the MTS assay is widely used as a surrogate measure of cell proliferation, it primarily reflects proliferation and therefore cannot distinguish reduced proliferation from altered metabolic state or reduced viability. Accordingly, future studies employing direct cell cycle or DNA synthesis markers, such as Ki67 immunostaining or BrdU incorporation, would provide a more definitive assessment of proliferative behaviour.

In summary, our findings support a model in which CREB3L1 contributes to pathological ECM gene regulation and altered cellular behaviour in glaucomatous lamina cribrosa cells. By linking ER stress-responsive transcriptional control to fibrotic remodelling at the optic nerve head, this work provides a foundation for future studies aimed at dissecting stress-adaptive pathways in glaucoma and evaluating their potential as therapeutic targets.

## 5. Conclusions

In conclusion, this study demonstrates that CREB3L1 expression is elevated in human lamina cribrosa cells derived from glaucomatous donors and that its suppression attenuates transcription of key extracellular matrix genes associated with fibrotic remodelling. The accompanying reduction in cell metabolic activity following CREB3L1 knockdown further suggests a role for this transcription factor in maintaining the activated cellular phenotype characteristic of glaucomatous LC cells.

These findings support a model in which CREB3L1 participates in stress-responsive transcriptional programmes that contribute to pathological ECM accumulation at the optic nerve head. However, the present data should be regarded as hypothesis-generating. Direct assessment of upstream stress signalling pathways, downstream protein-level changes, and validation in in vivo models will be essential to establish causality and therapeutic relevance.

Nevertheless, by identifying CREB3L1 as a potential molecular link between cellular stress responses and fibrotic gene regulation in glaucoma, this preliminary and hypothesis-generating work provides a foundation for future studies aimed at dissecting non–IOP-dependent mechanisms of optic nerve head damage and exploring novel targets for disease modification. Indeed, additional in vivo and mechanistic studies would strengthen the quality of this work.

## Figures and Tables

**Table 1:** Posterior sclera specimens from human donor eyes with diagnosed glaucoma (n = 3) and without glaucoma (non-glaucoma) (n = 3).

Donor ID	Age	Gender	Disease State	Eye Bank
41-02	88	F	Non-glaucoma	Central Florida (Tampa)
135-02	79	M	Non-glaucoma	Central Florida (Tampa)
297-02	87	F	Non-glaucoma	Central Florida (Tampa)
58-02	84	F	Glaucoma	Central Florida (Tampa)
600-02	86	M	Glaucoma	Central Florida (Tampa)
652-02	79	M	Glaucoma	Central Florida (Tampa)

**Table 2:** Primer sequences used for Quantitative Real-Time RT-PCR.

Gene Name	Forward	Reverse
*CREB3L1*	GAGACCTGGCCAGAGGATAC	GTCAGTGAGCAAGAGAACGC
*α-SMA*	AAAGCTTCCCAGACTTCCGC	TTCTTGGGCCTTGATGCGAA
*Col1A1*	TTCTGTACGCAGGTGATTGG	CATGTTCAGCTTTGTGGACC
*Fibronectin*	CAACACCGAGGTGACTGAGAC	GGACACAACGATGCTTCCTGAG
*18S*	GTAACCCGTTGAACCCCATT	CCATCCAATCGGTAGTAGCC

**Table 3:** Primary and secondary antibodies used for Western blotting.

Target Protein	Host Species	Target Species	Concentration	Supplier & Product Code	Secondary Antibodies
CREB3L1	Human	Human	1:500	ab137565 Abcam, Ballynew, Ireland	Goat anti-rabbit Sc-2030, Biotech, Santa Cruz, CA, USA)
β-actin	Mouse	Human	1:1000	Ab8226 (Abcam, Cambridge, UK)	Goat anti-mouse sc-2005 (Biotech, Santa Cruz, CA, USA)

## Data Availability

The original contributions presented in this study are included in the article. Further inquiries can be directed to the corresponding author.
